# N-Linked Glycosylation and Near-Infrared Spectroscopy in the Diagnosis of Prostate Cancer

**DOI:** 10.3390/ijms20071592

**Published:** 2019-03-29

**Authors:** Tijl Vermassen, Sander De Bruyne, Jonas Himpe, Nicolaas Lumen, Nico Callewaert, Sylvie Rottey, Joris Delanghe

**Affiliations:** 1 Department of Medical Oncology, Ghent University Hospital, 9000 Ghent, Belgium; tijl.vermassen@ugent.be (T.V.); sylvie.rottey@ugent.be (S.R.); 2 Department of Clinical Chemistry, Microbiology and Immunology, Ghent University, 9000 Ghent, Belgium; sander.debruyne@uzgent.be (S.D.B.); jonas.himpe@ugent.be (J.H.); 3 Department of Urology, Ghent University Hospital, 9000 Ghent, Belgium; nicolaas.lumen@ugent.be; 4 Unit for Medical Biotechnology, Inflammation Research Center, VIB–Ghent University, 9052 Zwijnaarde, Belgium; nico.callewaert@irc.vib-UGent.be; 5 Department of Clinical Chemistry, Ghent University Hospital, 9000 Ghent, Belgium

**Keywords:** Benign prostate hyperplasia, gleason score, infrared spectroscopy, N-glycosylation, prostate cancer

## Abstract

Background: Performing a prostate biopsy is the most robust and reliable way to diagnose prostate cancer (PCa), and to determine the disease grading. As little to no biochemical markers for prostate tissue exist, we explored the possibilities of tissue N-glycosylation and near-infrared spectroscopy (NIR) in PCa diagnosis. Methods: Tissue specimens from 100 patients (benign prostate hyperplasia (BPH), *n* = 50; and PCa, *n* = 50) were obtained. The fresh-frozen tissue was dispersed and a tissue N-glycosylation profile was determined. Consequently, the formalin-fixed paraffin-embedded slides were analyzed using NIR spectroscopy. A comparison was made between the benign and malignant tissue, and between the various Gleason scores. Results: A difference was observed for the tissue of N-glycosylation between the benign and malignant tissue. These differences were located in the fycosylation ratios and the total amount of bi- and tetra-antennary structures (all *p* < 0.0001). These differences were also present between various Gleason scores. In addition, the NIR spectra revealed changes between the benign and malignant tissue in several regions. Moreover, spectral ranges of 1055–1065 nm and 1450–1460 nm were significantly different between the Gleason scores (*p* = 0.0042 and *p* = 0.0195). Conclusions: We have demonstrated biochemical changes in the N-glycan profile of prostate tissue, which allows for the distinction between malignant and benign tissue, as well as between various Gleason scores. These changes can be correlated to the changes observed in the NIR spectra. This could possibly further improve the histological assessment of PCa diagnosis, although further method validation is needed.

## 1. Introduction

Prostate cancer (PCa) is the second most common cancer diagnosed worldwide, and the fifth most common cause of cancer-related death in males [1]. The prostate gland is made up of basal, luminal, and neuroendocrine cells embedded in a fibro-muscular stroma. Normal growth and development are regulated by several molecular pathways, for example, through the binding of the androgen and estradiol receptors [2]. In prostate disease, such as PCa, cancerous aberrations occur as a consequence of genetic mutations or changes in the tumor microenvironment [3]. Moreover, the basement membrane can be disrupted and the prostate specific antigen (PSA) can access the peripheral circulation [4].

Serum PSA (sPSA) is the most frequently used biomarker in the diagnosis of PCa [5]. However, the diagnosis of prostate cancer is finally based on the histology of the tissue obtained during the prostate biopsy, although the diagnosis and differentiation of the disease have become increasingly precise through improved risk stratification and advances in imaging techniques [6]. Moreover, various tissue biomarkers have emerged, which could allow for the stratification of PCa, and so assist in decision-making in post-biopsy low-risk patients and in post-radical prostatectomy patients [7,8,9]. Unfortunately, as most of these biomarkers focus on genetic alterations, further studies are needed to investigate both the clinical benefit of these new technologies and the financial ramifications [10].

It is therefore imperative to focus on the biochemical changes in the PCa tissue that could indicate whether or not a patient suffers from this malignancy. One possibility is to determine the aberrant N-glycosylation patterns of PCa. As the aberrant glycosylation of proteins is a fundamental characteristic of tumorigenesis and aggressive clinical behavior, focusing on cancer-specific PSA glycoforms could enhance PCa diagnosis [11,12]. Another method is the use of infrared spectroscopy. Near-infrared (NIR) spectroscopy is a nondestructive, label-free, and molecular vibrational spectroscopic technique that can provide complete information on the chemical and physical composition of biological samples by studying the vibrational transitions of the structures. In this technique, samples are irradiated with a NIR light. Some of this NIR light is absorbed by the molecules, bringing them to a higher vibrational state, which can be evaluated [13]. 

Both techniques focus on the chemical changes of molecules, which are an aberrant characteristic of various disease states, including cancer. The use of both techniques could alter the diagnostic landscape in PCa. The goals of this research are therefore to determine the diagnostic potential of tissue N-glycosylation and tissue infrared spectroscopy in the diagnosis and differentiation of PCa.

## 2. Results

### 2.1. Tissue Characteristics

The tissue characteristics (including weight, total prostate protein concentration, and prostate tPSA concentrations) are summarized in Table 1A. No differences were observed between the benign and malignant tissue based on weight, total protein concentration, total PSA concentration, or PSA ratio. On the other hand, a significantly lower PSA ratio was observed for high Gleason scores compared to low and intermediate Gleason scores (*p* = 0.0235; Table 1B).

### 2.2. Tissue Prostate Protein N-Glycosylation

**Figure 1:**
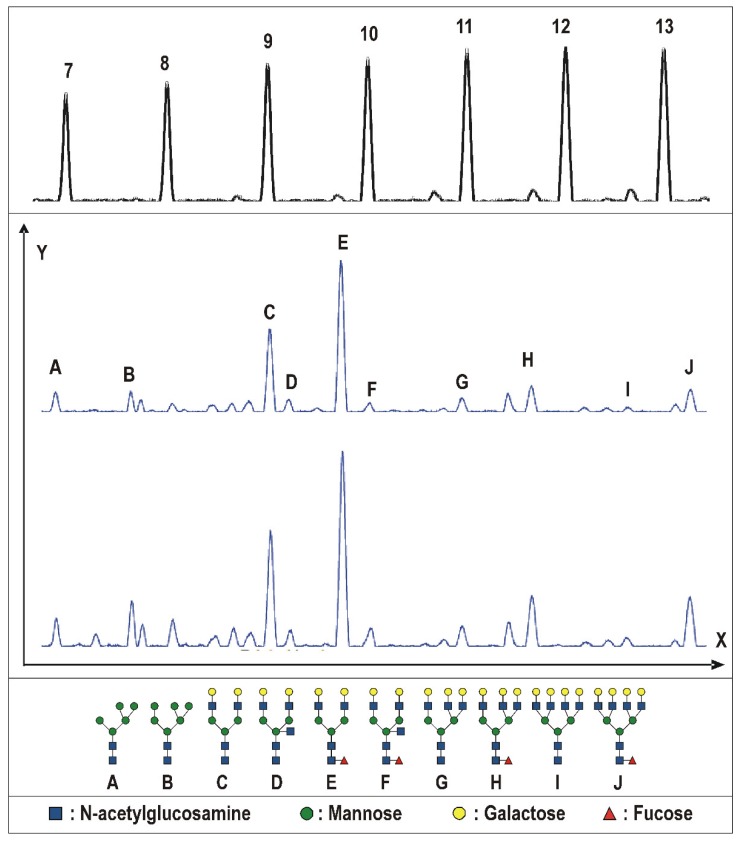
Desialylated urinary N-glycan profiles of prostate tissue specimens from a benign and malignant origin. The x-axis depicts the elution time; the y-axis represents the relative fluorescence units. Electropherograms have been depicted for the benign tissue (top) and malignant tissue (bottom). On top, a maltooligosaccharide ladder (dextran) is shown to determine the amount of structural units in each glycan structure (7–13). The identified glycosylation structures are shown in the figure (A–J). The glycan symbols are those suggested by the Consortium for Functional Glycomics (http://glycomics.scripps.edu/CFGnomenclature.pdf, accessed on: 20 February 2019). The relevant changes in N-glycan ratios are further illustrated in Figure 2.

**Figure 2:**
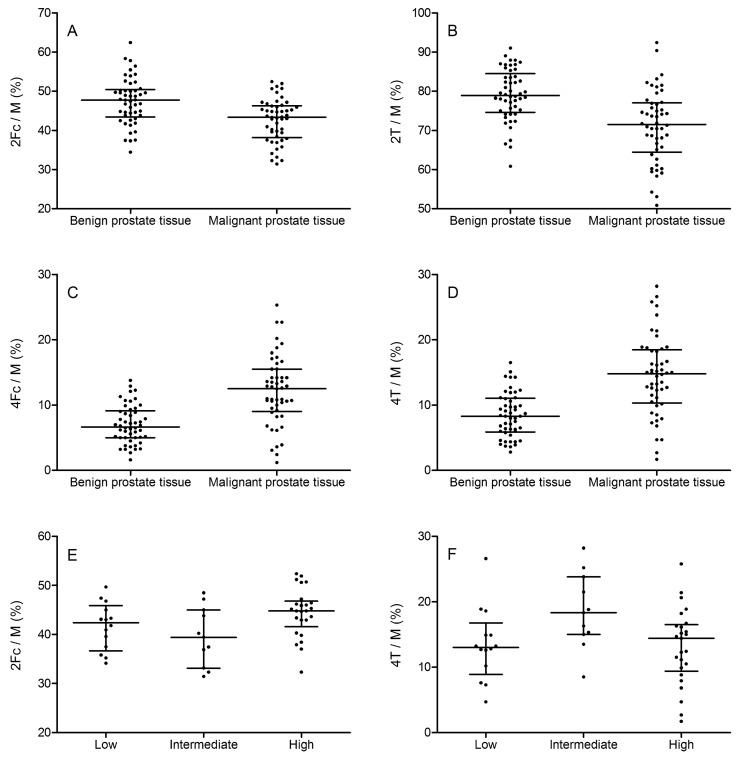
Differences in the N-glycan ratios. Plots are given for the differences in the N-glycan ratios between the various disease states. Comparisons are illustrated for the following: (**A**) 2Fc/M for benign versus malignant (*p* < 0.001); (**B**) 2T/M for benign versus malignant (*p* < 0.0001); (**C**) 4Fc/M for benign versus malignant (*p* < 0.0001); (**D**) 4T/M for benign versus malignant (*p* = 0.0300); (**E**) 2Fc/M for various Gleason scores (*p* < 0.001); and (**F**) 4T/M for various Gleason scores (*p* = 0.0327). 2Fc/M: total fucosylated biantennary structures on total multiantennary structures; 2T/M: total biantennary structures on total multiantennary structures; 4Fc/M: total fucosylated tetraantennary structures on total multiantennary structures; 4T/M: total tetraantennary structures on total multiantennary structures.

**Figure 3:**
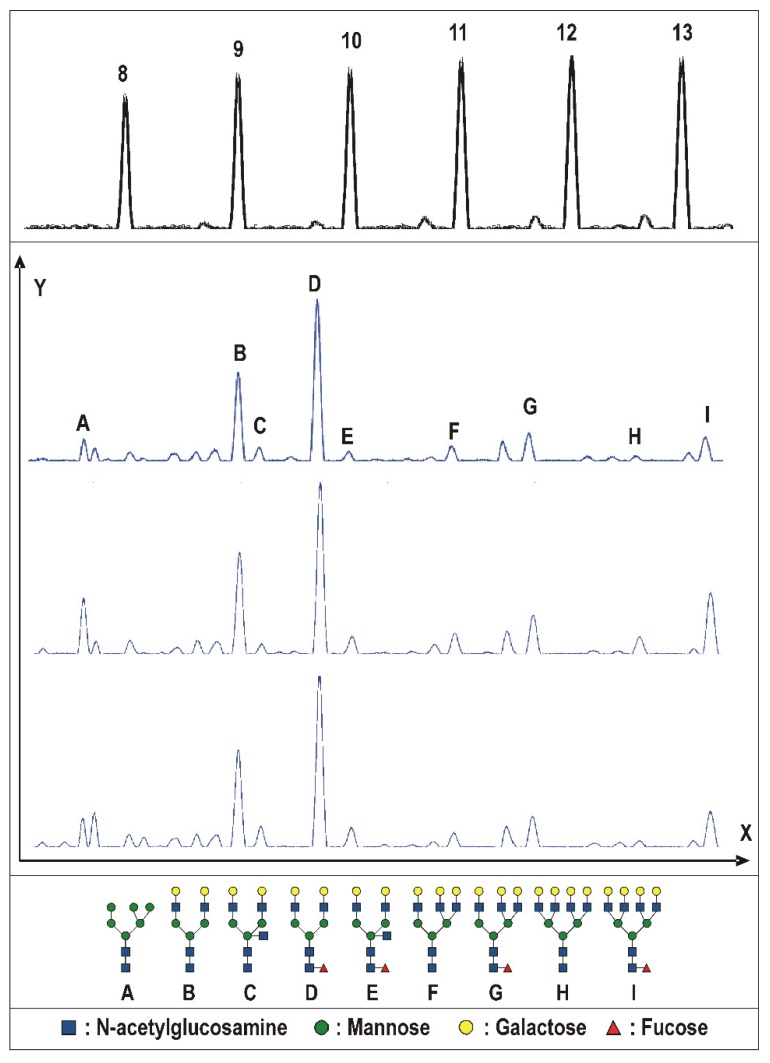
The desialylated urinary N-glycan profiles of the prostate tissue specimens from low, intermediate, and high Gleason scores. The x-axis depicts the elution time; the y-axis represents the relative fluorescence units. The respective electropherograms have been depicted for the low Gleason scores (3+3/3+4; top); intermediate Gleason scores (4+3; middle); and high Gleason scores (4+4 and higher; bottom). On top, a maltooligosaccharide ladder (dextran) is shown to determine the amount of structural units in each glycan structure (8–13). The identified glycosylation structures are shown in the figure (A–I). The glycan symbols are those suggested by the Consortium for Functional Glycomics (http://glycomics.scripps.edu/CFGnomenclature.pdf, accessed on 20 February 2019). The relevant changes in the N-glycan ratios are further illustrated in Figure 2.

The N-glycosylation profiles were obtained for all of the specimens. The representative N-glycosylation patterns for both the benign and malignant tissues are depicted in Figure 1. Significant differences were found for several N-glycosylation ratios, namely fucosylated biantennary structures, fucosylated tetraantennary structures, total amount of biantennary structures, and total amount of tetraantennary structures (all *p* < 0.0001; Figure 2A–D). Moreover, changes in the N-glycan profile were also able to differentiate between the Gleason scores (Figure 3). Herein, we noticed differences for the fucosylated biantennary structures (*p* = 0.0300) and for the total amount of tetraantennary structures (*p* = 0.0327; Figure 2E–F). Further analysis proved that both ratios were able to differentiate the intermediate Gleason scores from the low and high Gleason scores.

### 2.3. Tissue NIR Spectroscopy

**Figure 4:**
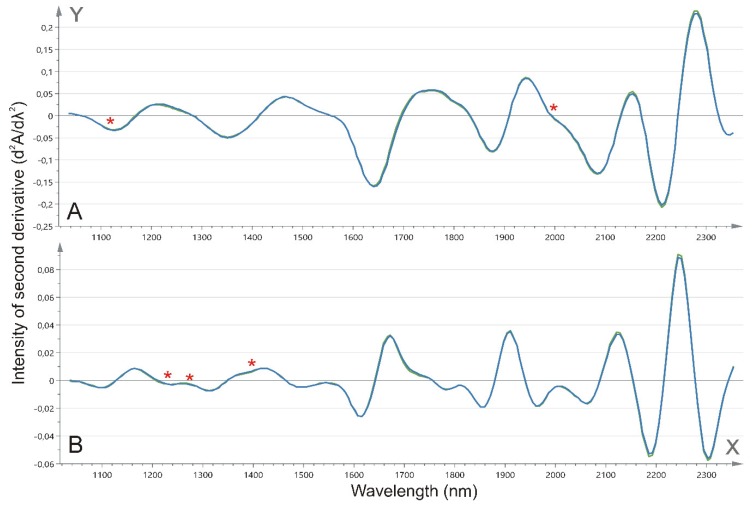
Mean near-infrared spectroscopy (NIR) spectra for benign versus malignant tissue. Mean NIR spectra from 1040 to 2340 nm for benign (green) and malignant tissue (blue). The x-axis depicts the wavelength (nm). The spectra are illustrated for (**A**) the first derivative (dA/dλ); and (**B**) the second derivative (d^2^A/dλ^2^). Nearly significant changes have been highlighted with an asterisk (*), and the spectral variation is further illustrated in Figure 5. A—absorbance; λ—wavelength.

**Figure 5:**
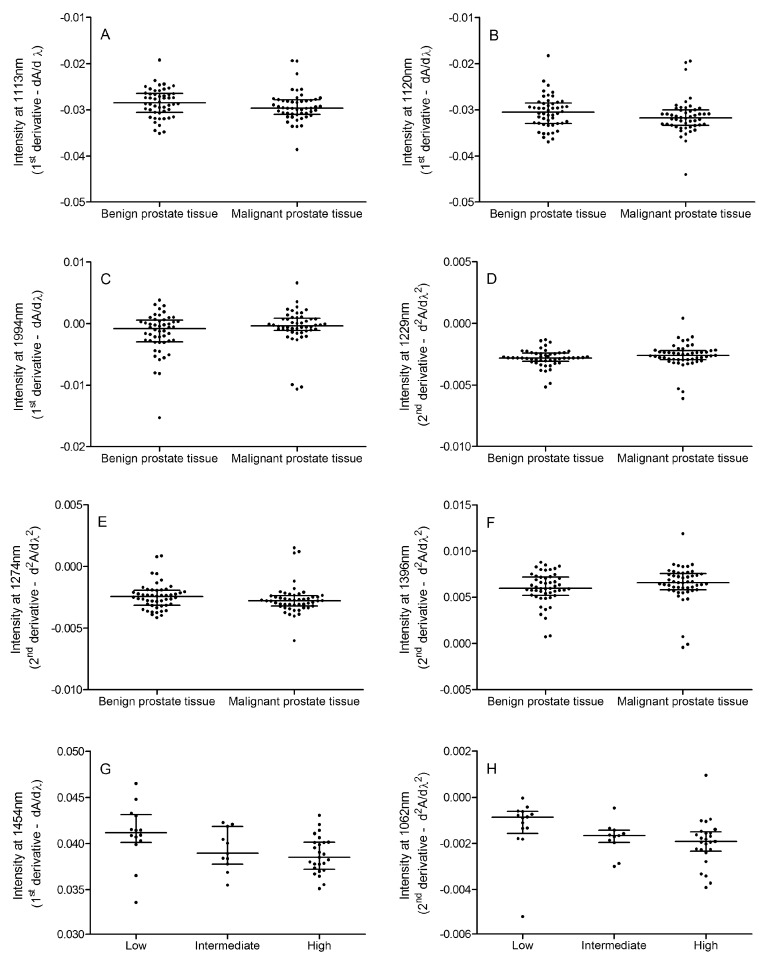
Differences in the NIR spectra. The plots are given for the differences in the NIR spectra between various disease states. Comparisons are illustrated for the following: (**A**) first derivative at 1113 nm for benign versus malignant (*p* = 0.0776); (**B**) first derivative at 1120 nm for benign versus malignant (*p* = 0.0667); (**C**) first derivative at 1994 nm for benign versus malignant (*p* = 0.0848); (**D**) second derivative at 1229 nm for benign versus malignant (*p* = 0.0994); (**E**) second derivative at 1274 nm for benign versus malignant (*p* = 0.0966); (**F**) second derivative at 1396 nm for benign versus malignant (*p* = 0.0776); (**G**) first derivative at 1454 nm for various Gleason scores (*p* = 0.0195); and (**H**) second derivative at 1062 nm for various Gleason scores (*p* = 0.0042).

Both the first and second derivative (Figure 4) obtained after the prostate tissue NIR spectroscopy were explored for their diagnostic properties. Although the spectra for the first derivative are mostly overlapping, nearly significant changes in the spectral intensities could be found between the benign and malignant prostate tissue at 1113, 1120, and 1994 nm (*p* = 0.0776, *p* = 0.0667, and *p* = 0.0848, respectively; Figure 5A–C). The intensities at 1113 and 1120 nm showed significant though weak correlations with the N-glycan ratio for the fucosylated biantennary structures (*r* = 0.219, *p* = 0.0283 and *r* = 0.227, *p* = 0.0234, respectively) although the latter could not be associated with any N-glycan ratio. Next, also, a trend toward significance at 1229 nm (*p* = 0.0994), 1274 nm (*p* = 0.0966), and 1396 nm (*p* = 0.0776) was observed for the comparison of the second derivative between the benign and malignant prostate tissue (Figure 5D–F). Again, the latter two intensities showed a weak and nearly significant correlation with the N-glycan ratio for the fucosylated biantennary structures (*r* = 0.191, *p* = 0.0569 and *r* = −0.178, *p* = 0.0758, respectively). Moreover, the cross-validation-based on the first derivative of the full spectral range resulted in a 86% correct classification for the benign versus malignant tissue. In this model, five out of 50 malignant tissue sections were misclassified as benign, while nine out of 50 benign tissue sections were misclassified as malignant.

**Figure 6:**
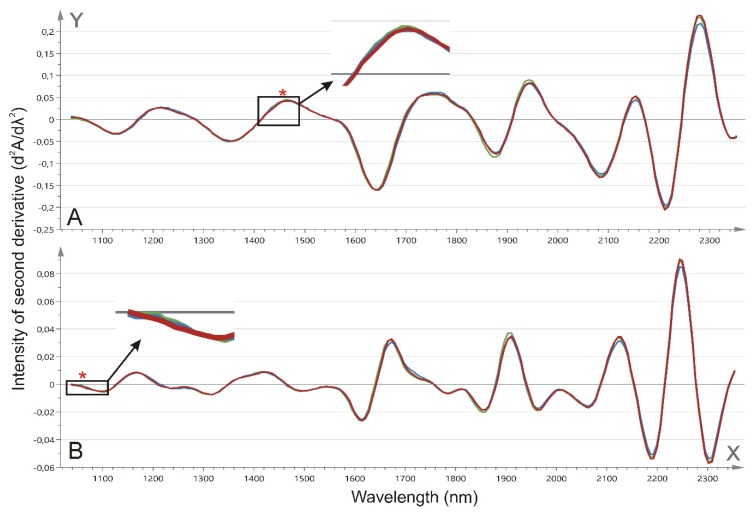
Mean NIR spectra for various Gleason scores. Mean NIR spectra from 1040 to 2340 nm for low (3+3/3+4; green), intermediate (4+3; blue), and high Gleason scores (4+4 and higher; red). The x-axis depicts the wavelength (nm). The spectra are illustrated for (**A**) the first derivative (dA/dλ) and (**B**) the second derivative (d^2^A/dλ^2^). Significant changes have been highlighted with an asterisk (*) magnified. The spectral variation is further illustrated in Figure 5. A—absorbance; λ—wavelength.

Next, the first and second derivative spectra were compared between the different Gleason scores (Figure 6). A significant difference was found for the first derivative intensity at 1454 nm (*p* = 0.0195), and for the second derivative at 1062 nm (*p* = 0.0042). Whereas the first could only differentiate between low and high Gleason scores, the latter enabled differentiation between low versus intermediate and high Gleason scores (Figure 5G–H). None of these spectral changes could be linked to the tissue N-glycan profile.

## 3. Materials and Methods

### 3.1. Tissue Specimens

A total of 100 prostate specimens were acquired from our Biobank—50 samples with only benign tissue (from BPH patients) and 50 samples with malignant PCa cells (from patients with PCa). All of the surgical procedures to obtain the prostate tissue occurred between August 2014 and March 2017. A small tissue fraction was taken for analysis. The absence or presence of malignant disease and the determination of the Gleason score (low = 3+3/3+4, intermediate = 4+3, and high = 4+4 and higher) in these tissue fractions were assessed on hematoxylin and eosin stained formalin-fixed paraffin-embedded (FFPE) slides. The FFPE slides were further used for NIR spectroscopy. All of the samples were stored at −80 °C until dispersion. The prostate specimens were weighed on an analytical balance (range 10–106 mg). Next, 3 mL of a 0.9% NaCl saline solution was added to the prostate specimen, which was homogenized for 20 s using an Ultra-Turrax homogenizer (IKA®-Werke GmbH & Co, Staufen, Germany). The dispersed tissue was transferred to a 5 mL tube and centrifuged at 870 g for 10 min. The supernatant was collected and aliquoted into the following three fractions: 500 µL for the biochemical analysis, 500 µL for the N-glycosylation analysis, and 1.5 mL as a spare fraction. A biochemical analysis was performed directly following the dispersion of the tissue specimens, whereas the other fractions were immediately stored at −20 °C. The N-glycosylation profile was determined within one week after storage.

The study was approved by the Ethics Committee of the Ghent University Hospital (21 January 2012; Belgian registration number: B670201214356).

### 3.2. Biochemical Analysis

The dispersed samples were analyzed for the total protein concentration and total PSA (tPSA) concentration. We used the Cobas^®^ 8000 modular P-analyzer series (Roche, Mannheim, Germany) to measure the total protein concentrations by means of the Total Protein Reagent Pyrogallol Red Method [14]. All of the reagents are commercially available (Instruchemie BV, Delfzijl, The Netherlands).

tPSA was assayed by means of electrochemiluminescence immunoassay on a Modular E170 analyzer series (Roche, Mannheim, Germany), and was standardized against the PreciControl Tumor markers 1 and 2 (Roche, Mannheim, Germany). All of the reagents are commercially available (Roche, Mannheim, Germany). The PSA ratio (mg/g protein) for each tissue specimen was determined by dividing the tPSA concentration by the total protein concentration.

### 3.3. Tissue Prostate Protein N-Glycosylation Profile

The determination of the tissue prostate protein N-glycosylation profile was performed using the on-membrane deglycosylation method, as previously described [15]. In short, the tissue prostate N-glycans were labeled with 8-aminopyrene-1,3,6-trisulphonic acid (Molecular Probes, Eugene, OR, USA), and were subsequently desialylated overnight at 37 °C by the addition of 2 µL 10 mM ammonium acetate pH 5.0 containing 40 mU of *Arthrobacter ureafaciens* α-2,3/6/8-sialidase. The end volume of the desialylated stock was 10 µL. Then, 2 µL of the desialylated N-glycan samples and a reference maltooligosaccharide ladder (dextran from *Leuconostoc mesenteroides*, Sigma-Aldrich, St. Louis, MO, USA) were analyzed with a multicapillary electrophoresis-based ABI3130 sequencer (Applied Biosystems, Foster City, CA, USA). The peaks were further analyzed with GeneMapper version 3.7 software (Applied Biosystems, Foster City, CA, USA), and the peak height intensities were normalized to the total intensity of the measured peaks. Different ratios were calculated.

### 3.4. Tissue NIR Spectroscopic Analysis

NIR spectroscopy was performed as an adaptation of the previous methods used by our group [16,17]. In short, the tissue sections were analyzed at room temperature by means of an extended InGaAs array technology enhanced NIR spectrometer (AvaSpecNIR256-2.5-HSC, Avantes, Apeldoorn, the Netherlands). Immersion oil was added onto the slides in order to eliminate the loss of resolution due to different refractive surfaces (glass versus air). Using an immobilized 50 mm integrating sphere (AvaSphere-50-LS-HAL-6-S1, Avantes), the spectra of light reflections were measured across the range of 1040 to 2340 nm.

The subsequent data analysis was performed via SIMCA software version 15.0 (MKS Data Analytics Solutions, Umeå, Sweden). Sample preprocessing via Standard Normal Variate (SNV), derivatives, and Savitzky–Golay (SG) smoothing was carried out in order to remove irrelevant light scatter and to standardize the obtained NIR spectra. The proposed sample processing methods work as follows: SNV eliminates both the baseline offset variations and multiplicative scaling effects, thus resulting in an accentuation of the structural differences, without interference of the baseline effects, such as differences in the sample density and the sample-to-sample measurement variations. Next, the application of spectrum derivatives enhances the resolution of the obtained spectra, which can allow for differentiation between overlapping bands. Two derivatives can be applied, namely: the first derivative in which the rate of change of absorbance (A) with respect to wavelength (λ) is examined (dA/dλ), and the second derivative, which measures the alterations in the rate of change of absorbance (d^2^A/dλ^2^). Finally, the enhancement of the signal-to-noise ratio without a loss of spectral details can be obtained via SG smoothing. Following the sample preprocessing, the diagnostic properties of the NIR spectroscopy on prostate tissue sections were evaluated. In this matter, a misclassification of samples was performed using a cross-validation procedure.

### 3.5. Statistical Analysis

The statistical analyses were performed with MedCalc Statistical Software version 13.3.1.0 (MedCalc Software, Ostend, Belgium) and GraphPad Prism version 4.7 (GraphPad Software Inc., La Jolla, CA, USA). Normal distribution of the subject groups was verified by the D’Agostino–Pearson test. The differences between the benign and malignant specimens were analyzed by means of unpaired Student’s *t*-tests or Mann–Whitney U tests. Changes between the Gleason scores were analyzed by one-way analysis of variance or the Kruskal–Wallis test. The correlation between the tissue N-glycosylation and spectral absorbance was determined using the Pearson *r* correlation. Finally, *p*-values of <0.05 were considered statistically significant.

## 4. Discussion

In our research, we focused on the determination of the diagnostic properties of N-glycosylation and NIR spectroscopy in the analysis of prostate tissue, as well as the possible association between both methods.

At first, the tissue characteristics of the benign and malignant prostate tissue were evaluated. The PSA ratio of malignant tissue appears to be slightly higher in comparison to the benign tissue, although no significant changes could be observed between the malignant and benign tissue. Nevertheless, interesting changes in the biochemical parameters were observed for a comparison between the different Gleason scores, as high Gleason scores (4+4 and higher) demonstrated a lower relative PSA concentration compared to low (3+3/3+4) and intermediate (4+3). To our knowledge, such a biochemical difference between the Gleason scores has not be demonstrated before in tissue, and could therefore possibly be used for tumor grading during the assessment of prostate biopsies.

Secondly, another biochemical marker has been explored, namely N-glycosylation, for its ability to differentiate benign from malignant tissue, and to distinguish different Gleason scores from each other. Data on prostate tissue N-glycosylation is however scanty [18]. Here, we found that an increase in the tetraantennary structures and a subsequent reduction in the biantennary structures (mostly the fucosylated form) occurred in the malignant tissue versus benign tissue. This is in line with other findings from our group, as we previously demonstrated a decrease in the fucosylation ratio of urine prostate proteins in PCa [12]. Unfortunately, our previously constructed urine biomarker was unable to differentiate the benign tissue from the malignant tissue.

Surprisingly, an increase in the fucosylated biantennary structures was noticed for high Gleason scores (4+4 and higher). This is contradictory to previous results, where we reported a decrease in the fucosylated biantennary structures with increasing Gleason scores [12]. As N-glycosylation is an important post-translational modification regulating protein trafficking [19], one possible explanation for the difference between the urine and tissue could be that proteins are differently glycosylated depending on their secretion pathway. On the other hand, we noticed a clear reduction in fucosylated biantennary structures for intermediate Gleason scores in comparison to low Gleason scores. This is in line with previously reported findings from our group. Moreover, to our knowledge, we are the first to indicate a change in a tissue biochemical parameter, which enables the differentiation between low (3+3/3+4) and intermediate (4+3) Gleason scores. The implementation of this technique could therefore be an important asset in PCa diagnosis, especially in the determination of disease risk. 

Lastly, we determined if NIR spectroscopy could contribute to the diagnosis of PCa, and if these changes could be correlated to the biochemical changes observed in the N-glycan profile.

Until now, various research groups have applied IR spectroscopy for the comparison between benign and malignant prostate tissue [20], and between different Gleason scores [21]. Mostly focusing on mid- and far-IR, very few research has, however, been conducted in the NIR spectral region. Recently, several fluorescent NIR imaging techniques have been described [22,23], although the approach described here is quite novel in the field of prostate tissue evaluation. Using NIR spectroscopy equipped with extended InGaAs array technology on hematoxylin and eosin stained FFPE slides, we were able to derive the NIR spectrum of both benign and malignant prostate tissue.

A comparison between the derivative spectra for benign and malignant tissue proved some interesting findings. For the first derivative, near significant changes were found at 1110–1120 nm and at 1990–2000 nm. Based on the overview described by Stuart [13], these wavelengths are associated with C–H second overtone stretching and a combination of O–H stretching (H_2_O). In comparison, we found several regions of interest for the second derivative, namely at 1225–1235 nm, 1270–1280 nm, and at 1390–1400 nm. The first two regions are probably associated with the C–H second overtone stretching, whereas the latter is associated with the OH first overtone stretching. It seems logical that these regions of interest allow for the distinction between benign and malignant prostate tissue, as most of these regions are correlated to the ratio of fucosylated biantennary structures found in prostate tissue, which contain a multitude of C–H bonds and O–H bonds. This would indicate that changes in the NIR spectra can allow for the determination of changes in the fucosylation ratio of proteins, which was indicated previously by Khajehpour et al. [24]. Caution is however advised, as NIR spectroscopy is not only able to visualize spectral variations in N-glycosylation, but also in O-glycosylation, which may be present in prostate tissue. Moreover, it has also been stipulated that there is a reduced water content in prostate cancer cells compared with normal prostate cells, and that the change of water-related peaks in the NIR spectra may be used as a diagnostic tool [25]. This could also account for the changes observed in the stretching of the O–H bonds between benign and malignant prostate tissue.

Similar results were obtained for the differentiation between the Gleason scores. We noticed a significant change in the NIR spectra for the first derivative at 1450–1460 nm, and for the second derivative at 1055–1065 nm, which are both linked to the stretching O–H bonds (first and second overtone, respectively). It has been reported previously that the FTIR spectra could allow for the differentiation between the Gleason scores. However, these studies have combined 3+4 and 4+3 Gleason scores [26,27], whereas the new guidelines for the diagnosis of PCa clearly stipulate that 3+4 Gleason scores must be considered as low risk in comparison to 4+3 scores [28]. To our knowledge, we are the first group to implement NIR spectroscopy in order to differentiate between low (3+3/3+4), intermediate (4+3), and high (4+4 and higher) Gleason scores. Additional NIR spectroscopy next to conventional microscopy could therefore improve the histological assessment of prostate tissue, as also indicated by Kim et al. [29] and Partsvania et al. [30].

However, our research faces several limitations that need to be addressed. Firstly, the number of tissue samples in our study might be too small to allow for a clear differentiation based on the NIR between tissues, especially for the differentiation between low-, intermediate-, and high-grade tumors. Secondly, the prostate gland is a very heterogeneous organ. It is therefore plausible that only limited changes can be noticed by focusing only on a small fraction of the prostate gland, whereas in previous research, we focused on the proteins derived from the entire prostate gland [12,15]. This is also applicable for the tissue sections, which can be very heterogeneous or even damaged [31]. It might therefore be advisable to acquire multiple tissue fragments from the same prostate to perform these analyses. In addition, an intrapatient comparison of sPSA, urine, and tissue N-glycosylation profiles could give a better understanding of the tumor development leading to damage in the tumor architecture by PSA. Thirdly, numerous confounding factors exist that could hamper these evaluations, such as inter-individual variation and differences in hormone exposure (e.g., androgen and androgen receptor expression) [32]. Further elaboration of all pre-analytical factors and method evaluation and optimization of both techniques is therefore highly recommended. In this matter, the evaluation of the androgen and androgen receptor expression in correlation to the tissue N-glycosylation/spectroscopy might be of interest, as androgens and androgen receptors are involved in both the growth and development of prostate tissue, as well as PCa progression [2]. Next, a comparison of the tissue N-glycosylation and tissue NIR to the current techniques used in daily practice is needed, as is an evaluation of their predictive and/or prognostic features during initial therapy for prostate cancer.

## 5. Conclusions

We were able to demonstrate biochemical changes in the N-glycan profile of prostate tissue, which allows for the distinction between malignant and benign tissue, as well as between various Gleason scores. Moreover, these changes can be correlated to the changes observed in the NIR spectrum. This could possibly further improve the histological assessment in PCa diagnosis, although further method validation is needed.

## Figures and Tables

**Table 1:** Tissue characteristics.

**(A) Benign Versus Malignant**					
**Characteristic**	**Benign Tissue**		**Malignant Tissue**		***p*-value**
**N**	50		50		
**Total prostate weight (g)**	55.5 (14.8–146.0)		49.0 (20.7–118.0)		0.5757
**Tissue prostate weight (mg)**	28.1 (10.4–106.2)		24.4 (11.4–61.5)		0.9917
**Total protein (g/L)**	0.29 (0.07–0.97)		0.25 (0.09–0.94)		0.7669
**PSA ratio (mg/ g protein)**	3.4 (0.2–24.0)		4.5 (0.1–21.5)		0.4179
(B) Between Different Gleason Scores *					
**Characteristic**	**Low**	**Intermediate**		**High**	***p*-value**
**N**	14	11		21	
**Total prostate weight (g)**	46.5 (34.0–95.0)	48.0 (28.5–86.0)		39.5 (20.7–118.0)	0.6953
**Tissue prostate weight (mg)**	24.7 (14.3–45.7)	30.7 (11.4–54.1)		23.5 (13.5–61.5)	0.7644
**Total protein (g/L)**	0.27 (0.18–0.85)	0.32 (0.09–0.73)		0.24 (0.10–0.94)	0.5110
**PSA ratio (mg/g protein)**	6.6 (1.1–20.2)	5.3 (0.8–21.5)		2.9 (0.1–7.0)	0.0235

*: Differentiation between low (3+3/3+4), intermediate (4+3) and high (4+4 and higher) Gleason scores. PSA—prostate specific antigen.
